# Proteome profiling of cerebrospinal fluid reveals biomarker candidates for Parkinson’s disease

**DOI:** 10.1016/j.xcrm.2022.100661

**Published:** 2022-06-21

**Authors:** Ozge Karayel, Sebastian Virreira Winter, Shalini Padmanabhan, Yuliya I. Kuras, Duc Tung Vu, Idil Tuncali, Kalpana Merchant, Anne-Marie Wills, Clemens R. Scherzer, Matthias Mann

**Affiliations:** 1 Department of Proteomics and Signal Transduction, Max Planck Institute of Biochemistry, Martinsried, Germany; 2 The Michael J. Fox Foundation for Parkinson’s Research, New York, NY, USA; 3 APDA Center for Advanced Parkinson Research, Harvard Medical School, Brigham and Women’s Hospital, Boston, MA, USA; 4 Precision Neurology Program, Harvard Medical School, Brigham and Women’s Hospital, Boston, MA, USA; 5 Department of Neurology, Massachusetts General Hospital, Boston, MA, USA; 6 Northwestern University Feinberg School of Medicine, Chicago, IL, USA; 7 Novo Nordisk Foundation Center for Protein Research, Faculty of Health Sciences, University of Copenhagen, Copenhagen, Denmark

**Keywords:** Parkinson’s disease, CSF, proteomics, mass spectrometry, DIA, biomarker, LRRK2

## Abstract

Parkinson’s disease (PD) is a growing burden worldwide, and there is no reliable biomarker used in clinical routines to date. Cerebrospinal fluid (CSF) is routinely collected in patients with neurological symptoms and should closely reflect alterations in PD patients’ brains. Here, we describe a scalable and sensitive mass spectrometry (MS)-based proteomics workflow for CSF proteome profiling. From two independent cohorts with over 200 individuals, our workflow reproducibly quantifies over 1,700 proteins from minimal CSF amounts. Machine learning determines OMD, CD44, VGF, PRL, and MAN2B1 to be altered in PD patients or to significantly correlate with clinical scores. We also uncover signatures of enhanced neuroinflammation in LRRK2 G2019S carriers, as indicated by increased levels of CTSS, PLD4, and HLA proteins. A comparison with our previously acquired urinary proteomes reveals a large overlap in PD-associated changes, including lysosomal proteins, opening up new avenues to improve our understanding of PD pathogenesis.

## Highlights

• Reproducible and scalable MS-based proteomics workflow for proteome profiling of CSF
• Quantification of over 1,700 proteins collected from more than 200 individuals
• Lysosomal and immune-related biomarker signatures specific to LRRK2 G2019S carriers
• Overlapping PD- and LRRK2-associated alterations in CSF and urine proteomes

## Graphical abstract

**Figure:**
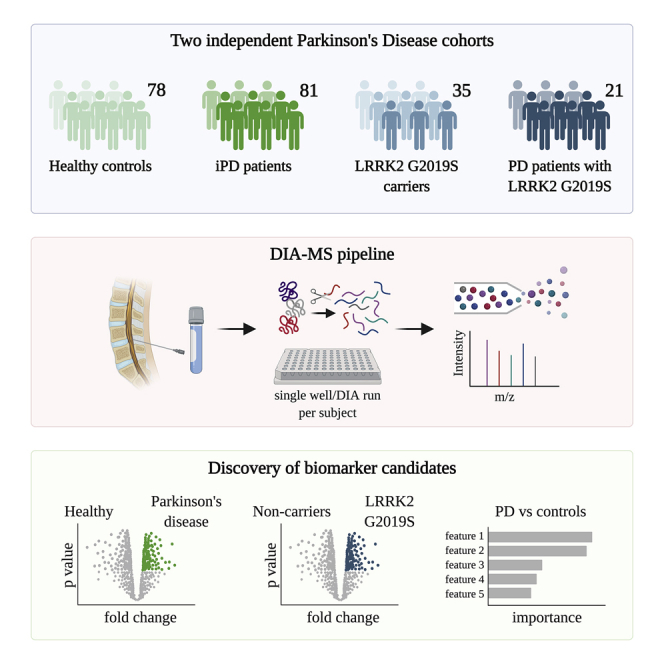

## Teaser

Karayel et al. present a scalable, sensitive, and reproducible mass spectrometry-based proteomics workflow for profiling of cerebrospinal fluid and demonstrate its promise for biomarker discovery for idiopathic and familial forms of Parkinson’s disease.

## Introduction

Parkinson’s disease (PD) is the second most common neurodegenerative disease, affecting millions of people worldwide and having a strikingly increased incidence with age.^1^^,^^2^ The hallmark pathology of PD is well characterized as α-synuclein aggregates and dopamine neuronal loss;^2^ however, molecular events that trigger PD are not fully understood. As a result, all current treatments only target symptoms, without slowing or reversing disease progression.^3^ Thus, detecting protein level alterations in PD could provide insight into the underlying mechanism of disease and aid in the development of novel therapeutics.

The majority of PD cases are idiopathic, while for some a genetic linkage is apparent.^3^, ^4^, ^5^ Mutations in the LRRK2 gene, most commonly G2019S, are the most frequent genetic cause of autosomal dominant PD.^6^^,^^7^ Importantly, all PD-associated mutations activate leucine-rich repeat kinase 2 (LRRK2) kinase activity, offering a promising therapeutic target for PD by inhibiting this function.^7^, ^8^, ^9^ Better understanding of LRRK2-PD pathophysiology and discovery of related biomarkers would facilitate LRRK2-targeted therapies. It is also essential to determine whether pathogenic mechanisms associated with LRRK2-PD may be present at the prodromal stage in non-manifesting LRRK2 mutation carriers and in a subset of idiopathic PD.

Cellular and animal model studies indicate that LRRK2 is involved in the regulation of several pathophysiologic processes, including autophagy, endolysosomal membrane/vesicular trafficking, and immune responses, with at least some of the effects mediated through phosphorylation of a subgroup of Rab GTPase.^10^, ^11^, ^12^, ^13^ Whereas cell biology research on molecular pathways affected by LRRK2 is critical for insights into disease mechanisms, a complementary approach would be to examine molecular changes in biospecimens from deeply phenotyped LRRK2 and idiopathic PD cohorts compared with controls. We thus reasoned that an unbiased, mass spectrometry (MS)-based proteomics analysis of cerebrospinal fluid (CSF) from heathy controls, PD individuals with and without LRRK2 G2019S mutation and non-manifesting LRRK2 G2019S carriers could uncover much-needed biomarkers. Compared with other biofluids, CSF should reflect the disease-related pathology of the brain and spinal cord more accurately.^14^^,^^15^

MS-based proteomics is a very powerful technology for detecting differences in protein abundance levels in healthy individuals and patients, and thus, in principle, an ideal tool for biomarker discovery. However, proteomic analysis of CSF has been challenging due to low protein concentration combined with the high dynamic range of protein abundances, resulting in low quantification precision, throughput, and limited proteome depth.^16^, ^17^, ^18^ Recent advances in the proteomics field, from automated sample preparation to more sensitive MS instrumentation, data acquisition methods such as data-independent acquisition (DIA) and processing software, have enabled substantial proteome coverage and precise quantitation in single LC-MS runs.^19^, ^20^, ^21^, ^22^, ^23^ Our group has combined these advances into a streamlined and highly reproducible workflow resulting in a large number of consistently measured and biologically meaningful proteome changes in various biofluid/tissue specimens in a variety of clinical cohorts.^16^^,^^17^^,^^24^, ^25^, ^26^, ^27^

In this study, we extended our pipeline of biomarker discovery to analyze more than 200 CSF samples from two independent cohorts in which we detected over 1,700 proteins from minimal CSF sample amounts. By employing co-variate (ANCOVA) analysis and machine learning, we identified unique protein signatures whose abundance was specifically changed in PD patients versus healthy controls and LRRK2 G2019S carriers versus non-carriers. Our study demonstrates that modern MS-based proteomics is a powerful technology for biomarker discovery in biofluids. It also provides potential biomarkers of PD as well as insights into biological pathways associated with PD and/or LRRK2 mutations.

## Results

### Overview of PD cohorts for CSF proteome profiling

To investigate how PD affects the CSF of patients and to identify potential biomarkers, we employed the “rectangular” biomarker discovery strategy, which aims to discover discriminating proteome signatures using rather large sample sizes in both discovery and validation cohorts.^16^^,^^17^^,^^24^^,^^28^^,^^29^ Applying this approach, we analyzed CSF samples from 215 individuals from two independent cohorts including 113 healthy controls (HC) and 102 PD patients. The first cohort consisted of 94 CSF samples from the Harvard Biomarkers Study (hereinafter referred to as the HBS cohort).^30^, ^31^, ^32^, ^33^ The second cohort was a subset of biobanked CSF samples from the Michael J. Fox Foundation for Parkinson’s Research (MJFF)-funded *LRRK2* Cohort Consortium (hereinafter referred to as the LCC cohort). More information about the cohorts is summarized in Table 1, Table S1 and Figure S1.

**Table 1:** Demographics of all participants

LCC cohort	LRRK2–/PD– (n = 31)	LRRK2+/PD– (n = 35)	LRRK2–/PD+ (n = 34)	LRRK2+/PD+ (n = 21)
Sex (male/female)	13/18	19/16	23/11	10/11
Age at collection, mean (SD)	54.2 (13.9)	52.1 (15.2)	57.2 (11.2)	62.2 (10.8)
Age at onset, mean (SD)	n/a	n/a	51.1 (11.4)	53.9 (11.4)
UPDRS III, mean (SD)	n/a	n/a	25.9 (9.4)	23.1 (15.6)
MOCA score, mean (SD)	27.3 (2)	26.2 (2.5)	26.5 (3)	24.7 (3.9)

**HBS cohort**	**PD- (n=47)**	**PD+ (n=47)**		

Sex (male/female)	29/18	34/13		
Age at collection, mean (SD)	56.4 (11.3)	62.8 (8.9)		
Age at onset, mean (SD)	n/a	58.4 (8.4)		
GBA (mut/WT), mean (SD)	0/47	6/41		
LRRK2 (mut/WT), mean (SD)	0/47	1/46		
UPDRS total, mean (SD)	n/a	37.5 (14.3)		
UPDRS III, mean (SD)	n/a	22.1 (14.1)		
MMSE score, mean (SD)	28.4 (1.8)	28.3 (1.6)		

### Proteomic characterization of CSF samples

**Figure 1:**
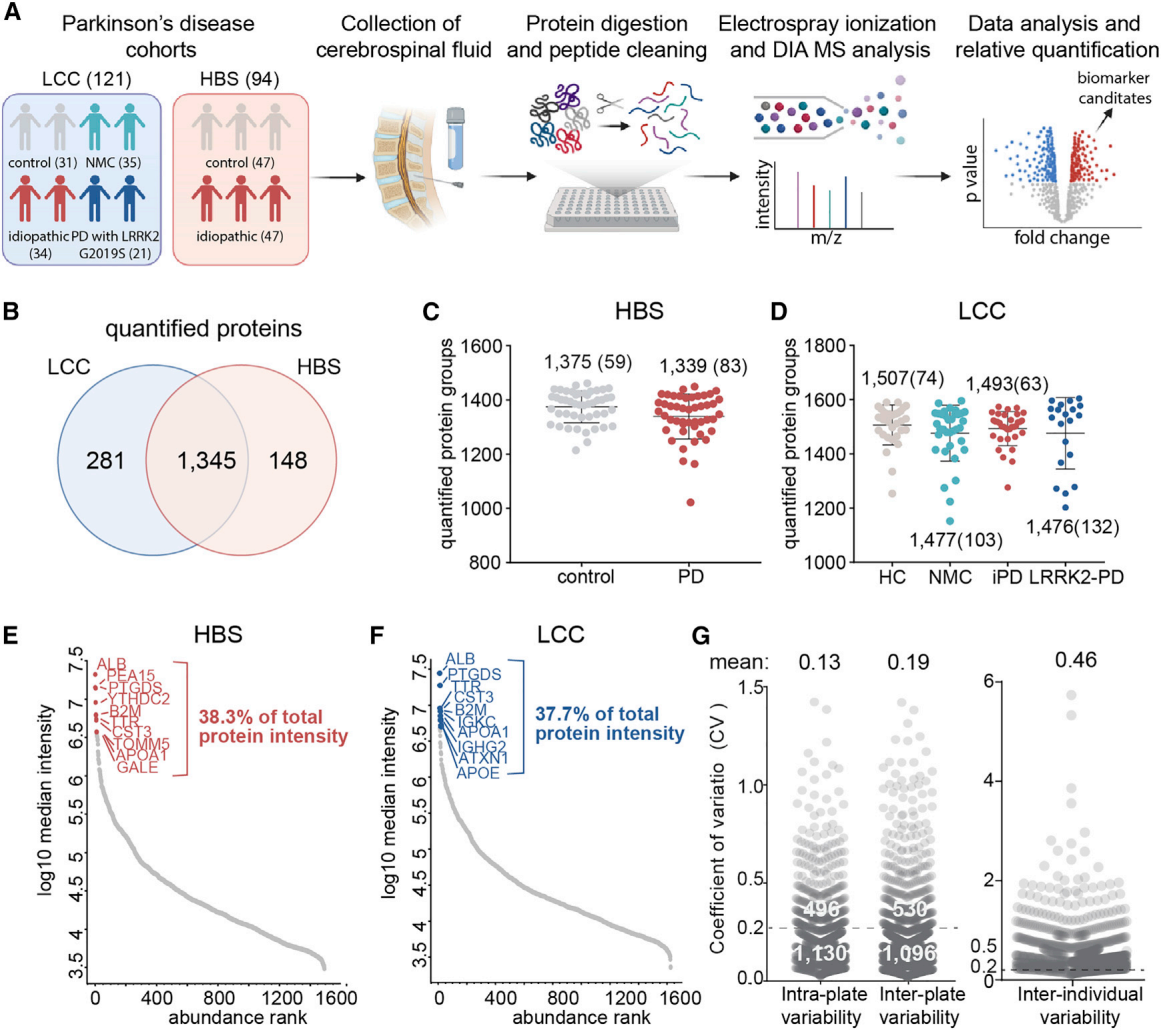
MS-based proteomic analysis of two independent CSF PD cohorts (A) Overview of the CSF proteomic workflow. CSF samples were prepared in 96-well plates using an automated liquid-handling system and analyzed by LC-MS/MS using data-independent acquisition (DIA). The total number of subjects per cohort group is shown. (B) A total of 1,345 proteins were consistently quantified in both cohorts. (C and D) Number of proteins identified and quantified with a 1% false discovery rate (FDR) in each sample in the HBS (C) and LCC (D) cohorts. Numbers indicate mean and standard deviation (SD). (E and F) Proteins identified in the HBS (E) and LCC (F) cohorts were ranked according to their MS signals. The top 10 most abundant proteins are labeled, and their relative contribution to the total protein intensity is indicated. (G) Quantification precision assessed by calculating the intra- and inter-plate (between repeated measurements of the same sample) and inter-individual coefficients of variation (CVs) of all proteins. Number of proteins with a CV below and above 20% and mean CV values are shown.

We have developed a robust, automated, and high-throughput MS-based proteomics workflow using a data-independent acquisition (DIA) strategy to perform proteome profiling of minimal amounts of CSF^21^^,^^24^^,^^34^ (Figure 1A). This strategy has previously resulted in an unprecedented depth at high data completeness in CSF and revealed biologically meaningful proteome changes across multiple independent Alzheimer’s disease cohorts.^24^ Here, we applied our workflow to discover proteome changes in the CSF of PD patients with or without the disease-associated G2019S mutation in *LRRK2*. To maximize proteome depth and coverage, we generated cohort-specific hybrid spectral libraries by merging three sub-libraries: (1) a library constructed by data-dependent acquisition (DDA) consisting of 24 fractions of pooled CSF samples; (2) another DDA library consisting of eight fractions of extracellular vesicles enriched from pooled CSF samples; and (3) a direct-DIA library generated from the DIA analysis of all samples (see STAR Methods). Matching to cohort-specific hybrid libraries of 5,418 and 3,167 proteins yielded 1,493 and 1,626 proteins in total for the HBS and LCC cohorts, respectively, with more than 1,300 in common (Figure 1B). On average, we quantified 1,357 (HBS) and 1,481 (LCC) protein groups per neat CSF sample in single runs with 1,290 (HBS) and 1,440 (LCC) protein groups quantified in more than 70% of the samples (Figures 1C, 1D, S1J, S1K, and Table S2). Our DIA workflow resulted in a much deeper proteome coverage compared with previous studies applying a similar single-run DIA strategy without compromising throughput.^28^^,^^29^^,^^35^ The quantified protein intensities spanned over four orders of magnitude (Figures 1E and 1F). The top 10 most abundant proteins alone contributed around 38% to the total CSF proteome signals, illustrating the analytic challenges (Figures 1E and 1F).

We further classified the identified proteins based on their human protein atlas (HPA) annotation^36^ as secreted from the cell, intra-cellular or in the cellular membranes (note that many proteins are assigned to several compartments). In total, 94% of identified proteins carried at least one annotation, of which 36% were secreted proteins, while 70% were intra-cellular and 36% membrane-spanning proteins (Figure S2A). Furthermore, the majority of quantified proteins were annotated to be enriched in brain and liver, in line with the fact that CSF is the extracellular fluid of the CNS and derived from blood plasma, which contains many proteins synthesized in the liver (Figure S2B). In summary, using small CSF volumes, we obtained a very high CSF proteome coverage for single-run analysis, thus providing a promising basis for the discovery of biomarkers in PD.

### Assessment of quantification precision and sample quality

To assess quantification precision in our study, we investigated intra- and inter-assay variabilities of our automated workflow by repeated measurements of a pooled CSF sample (Figure S2C). This analysis revealed high technical reproducibility with 13% intra-plate and 19% inter-plate coefficients of variation (CVs). Around 1,100 had inter- and intra-assay CVs below 20% and 1,500 below 50% (Figures 1G, S2C and S2D). The inter-individual biological variability between subjects is much larger, with only 10% of all proteins having a CV below 20% (Figure 1G). This demonstrates that the technical variability of our assay is much smaller than the biological variability—an important pre-condition for the successful discovery of PD-specific proteome signatures and potential biomarkers in CSF.

**Figure 2:**
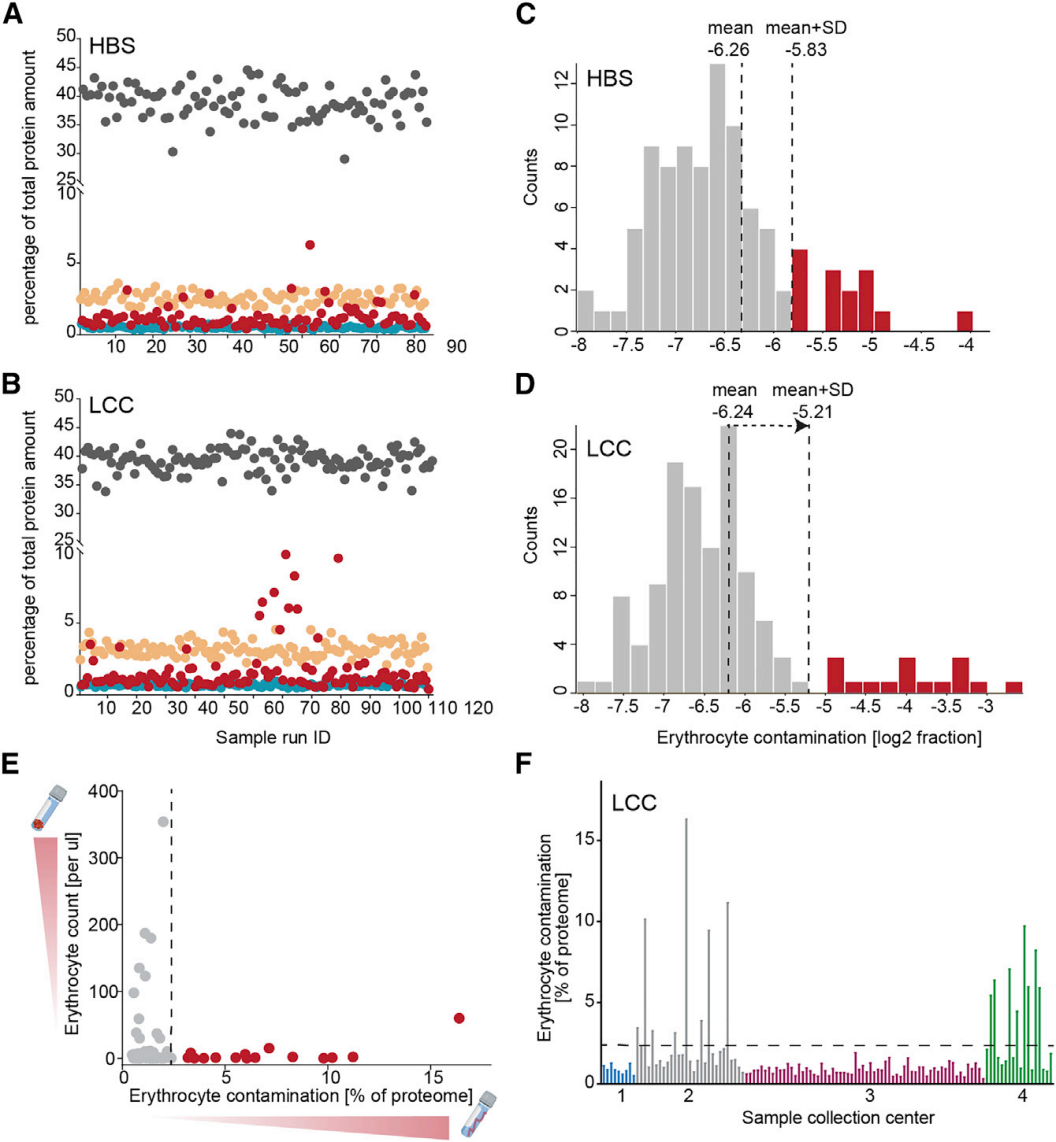
Quality assessment of two independent CSF PD cohorts (A and B) Assessment of study quality by determining the percentage of the summed intensity of the proteins in the respective quality marker panel and the summed intensity of all proteins in the HBS (A) and LCC (B) cohorts. Erythrocyte-specific protein panel (red), platelet marker panel (turquois), coagulation marker panel (orange), and the top 10 most abundant protein panel (dark gray) are included in these analyses. The proteins in each quality marker panel are listed in Table S3. (C and D) Histograms of log2 ratios of the summed intensity of the erythrocyte-specific proteins and the summed intensity of all proteins in the HBS (C) and LCC (D) cohorts. A sample was flagged for potential contamination and removed from further analysis if the ratio differed more than one SD from the mean of all samples within the cohort. (E) Comparison of erythrocyte counts in CSF following sample collection and degree of erythrocyte contamination as determined by MS-based proteomics of all LCC cohort samples. Samples colored in red were excluded from further analysis based on the distribution shown in (D). (F) Grouping samples in the LCC cohort for four sample collection centers demonstrated a high degree of contamination with erythroid-specific proteins for study centers 2 and 4, whereas there was no indication of this in study centers 1 and 3.

Inconsistent sample collection and handling may result in systematic bias and hamper the discovery of true biomarkers. To ensure that the quantified changes in CSF proteins are due to disease-related pathological alterations, we assessed the quality of all samples according to previously established quality marker panels for coagulation-related proteins, platelets, and erythrocytes to identify samples with potential issues in pre-analytical processing^16^^,^^17^^,^^37^ (Figures 2A and 2B). This revealed high sample-to-sample variability for the degree of contamination with our erythroid-specific marker panel, presumably due to puncture-related contamination with blood (Figures 2A and 2B). We flagged 14 samples (8 PD, 6 HC) from the HBS cohort and 17 samples (4 PD, 13 HC) from the LCC cohort for erythrocyte contamination and removed them from further analysis (Figures 2C and 2D). In total, proteomes of 182 subjects fulfilled the quality criteria and were used for statistical analysis.

We found no correlation between erythrocyte counts and the number of erythrocyte-specific proteins in the CSF (Figure 2E). Our samples were centrifuged following collection to remove cells and debris, explaining why initially high erythrocyte counts did not affect the proteome measurements. Hemolysis occurring during collection, however, would be visible only in the proteome and not in erythrocyte counts. Sorting the samples by study center revealed a systematic bias in the sample taking and processing procedure and identified two centers with high pre-analytical variation and a corresponding high degree of proteome contamination with erythroid-specific proteins. This further emphasizes the importance of standardized sample collection and processing procedures to minimize or avoid such biases and the advantage of unbiased proteomics to flag and remove such problematic cases.

We additionally further excluded a single sample from the HBS cohort that clustered far away from all other samples in a principal-component analysis (PCA) and a single sample from the LCC cohort that was an outlier in proteome depth. Our subsequent bioinformatics analysis was based on 79 samples from the HBS cohort and 103 subjects from the LCC cohort to determine the impact of disease manifestation and LRRK2 mutation status on the CSF proteome (Figures S2E and S2F).

### PD-related proteome alterations in CSF and machine-learning-based classification of PD patients and controls

**Figure 3:**
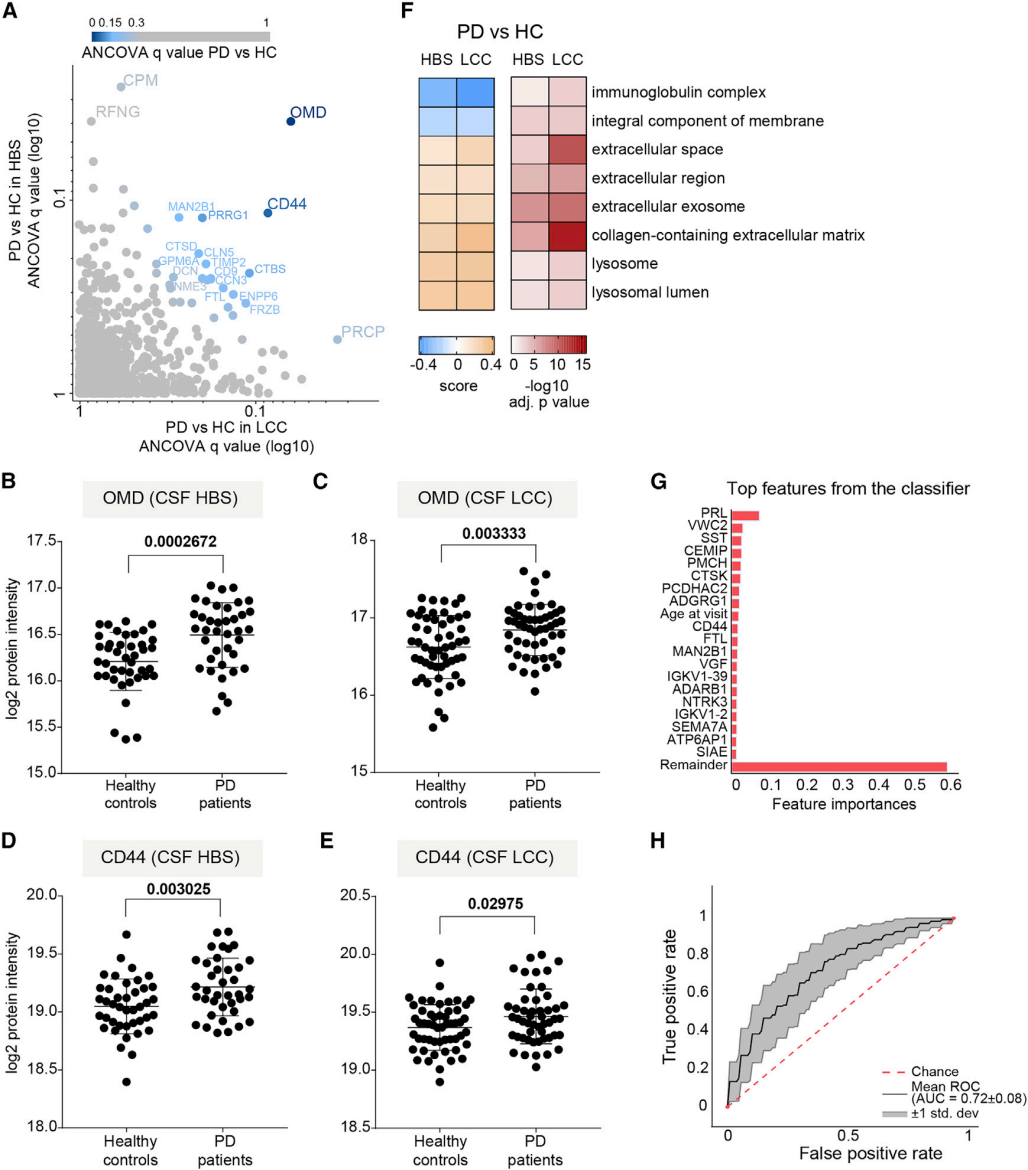
PD-related alterations in CSF proteome and ML-based classification of PD status (A) Correlation of ANCOVA q values of all proteins quantified in the CSF of PD patients compared with controls in the HBS and LCC cohorts. Color gradient is based on the mean of ANCOVA q values (PD versus HC) obtained in the LCC and HBS cohorts. (B and C) Osteomodulin (OMD) protein intensity (log2) distribution in controls and PD patients of the HBS (B) and LCC (C) cohorts. We applied an unpaired t test and the resulting p value is shown. (D and E) CD44 protein intensity (log2) distribution in controls and PD patients of the HBS (D) and LCC (E) cohorts; the corresponding p values from unpaired t test are shown. In panels B-E, lines indicate mean and SD. (F) Annotation enrichment of GO terms using the PD versus HC fold changes (5% FDR). All significantly enriched GO terms that were common in both cohorts are displayed. Terms with positive enrichment scores are enriched in PD over HC and vice versa. (G) Feature importance of the top 20 most important features used to distinguish PD+ versus PD–individuals. (H) ROC curve and corresponding AUC statistics in 5-fold cross-validation repeated 10 times using the XGBoost-based model to classify PD versus HC based on protein panel in (G). Random performance is indicated by the dotted diagonal red line for comparison. Gray area represents the SD from the mean ROC curve. Blue lines show the values for a total of repeats with five stratified train-test splits.

To investigate alterations in the CSF proteome of PD patients compared with controls, we performed an ANCOVA, considering age, sex, and *LRRK2* mutation status as confounding factors. We also included the different study centers as a confounding factor for the LCC cohort. For the HBS cohort, we further included the *GBA* mutation status. At 5% false discovery rate (FDR), we identified three significantly regulated proteins (CPM, OMD, and RFNG) in the CSF of PD patients compared with controls in the HBS and one (PRCP) in the LCC cohort (Figure 3A and Table S4). Osteomodulin (gene name: OMD) and the cell surface marker CD44 almost reached statistical significance in both cohorts (OMD: q values of 0.063 in LCC and 0.039 in HBS; CD44: q values of 0.08 in LCC and 0.12 in HBS; Figure 3A). One reason for the small overlap between the cohorts could be a less stringent and inconsistent sample collection protocol in the multi-center LCC study. Yet, the osteomodulin and CD44 proteins were robustly quantified in both cohorts and detected with three (CD44) and seven (OMD) peptides in all samples (Table S4). The levels of both proteins were significantly elevated in PD patients with mean fold changes of 1.22 (OMD) and 1.12 (CD44) in the HBS and 1.17 (OMD) and 1.07 (CD44) in the LCC cohort (Figures 3B–3E). These results demonstrate that the rectangular strategy was able to distinguish PD-related alterations comprising a few proteins differentially present in PD patients compared with controls from cohort-specific effects in the quantified CSF proteome, even in cohorts constrained by biases such as less stringent and inconsistent sample collection.

To examine whether PD affects particular cellular compartments and biological networks in CSF, we performed a gene ontology (GO) annotation enrichment analysis using the mean fold changes of PD versus HC.^38^ The proteins elevated in the PD samples compared with the controls were enriched for lysosomal-related terms, further supporting the emerging role of lysosomes and mounting evidence for lysosomal dysregulation and associated α-synuclein aggregation in PD^13^^,^^39^^,^^40^ (Figure 3F).

Motivated by the presence—but small number—of commonly altered proteins in both cohorts, we tested how well machine learning (ML) could discriminate PD patients from controls by using the recently introduced open-source tool OmicLearn.^41^ ML approaches strongly benefit from a large number of samples, which prompted us to combine the HBS and LCC cohorts to identify reliable signatures. Using the Extra-Trees package, the 35 most discriminating proteins for training the model were selected in each training iteration, and they were ranked by the classifier according to their feature importance (Figure 3G). Interestingly, among them, prolactin (gene name: PRL), was the most important feature based on 50 training iterations (5 splits, 10 repeats). Although prolactin was not significantly regulated in either cohort, its release is known to be suppressed in PD patients taking dopaminergic medications.^42^ PRL did not correlate with levodopa equivalent daily doses (LEDD) provided to all PD subjects in the HBS cohorts (Figure S3E). Further promising candidates to classify PD versus controls were CD44, VGF, and—in agreement with the lysosomal pathway enrichment—lysosomal proteins cathepsin K (CTSK) and MAN2B1. When these features to train XGBoost were used, an ensemble-tree-based model, with our cross-validation scheme, the mean area under the curve (AUC) of the receiver operating characteristic (ROC) curve was 0.72 ± 0.08. The AUC is a frequently used measure of performance of the classification model and ranges from 0 (all predictions are wrong) to 1 (all predictions are correct). The sensitivity, which is the rate of correctly classified PD patients, and specificity, which is the rate of correctly classified PD-negative individuals, were 67% and 66%, respectively (Figure 3H)*.* Taken together, our CSF proteome data, when combined with ML algorithms, classified disease status and, more importantly, identified several promising PD-associated proteins, opening up interesting leads for future studies.

### Impact of the pathogenic LRRK2 G2019S mutation on the CSF proteome

**Figure 4:**
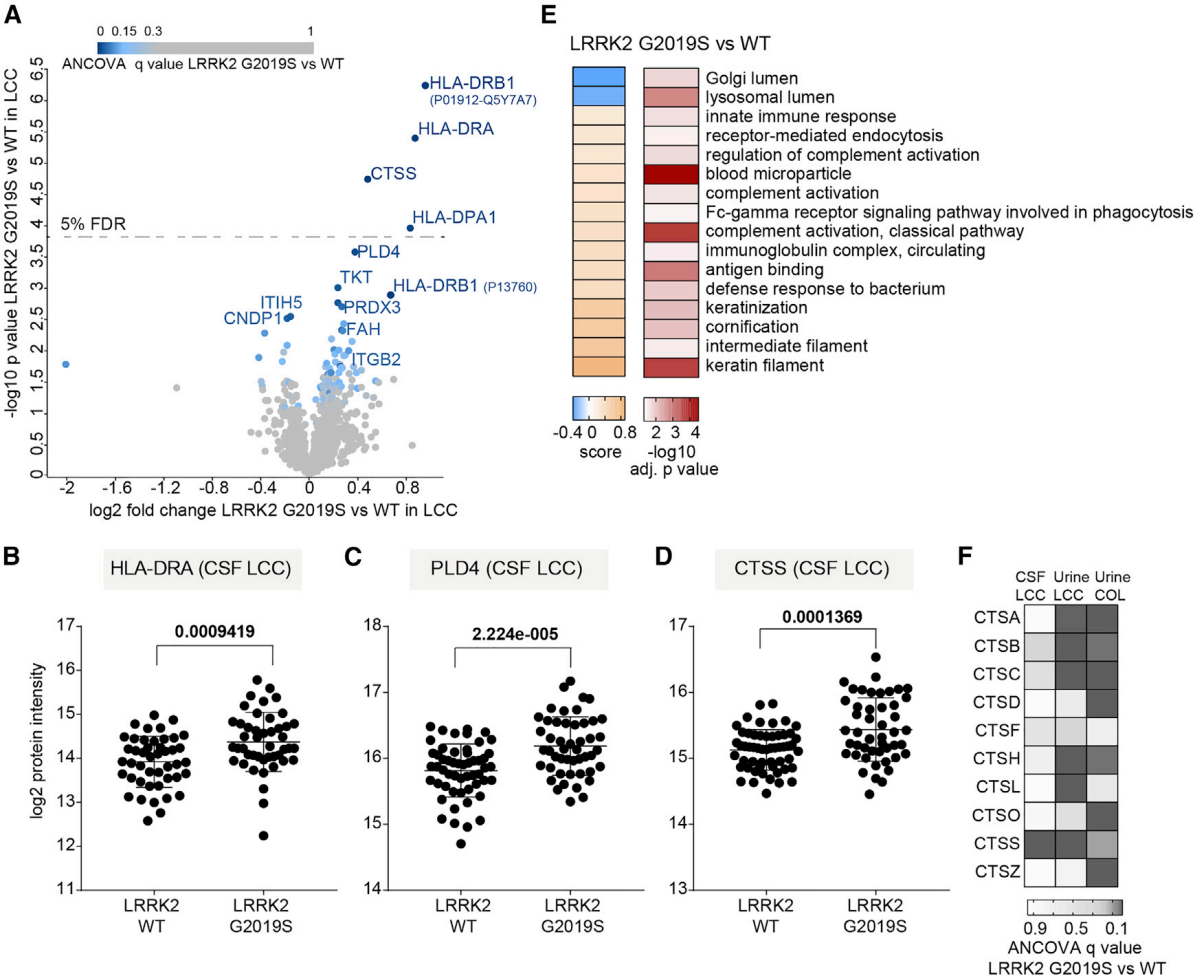
Effect of the pathogenic LRRK2 G2019S mutation on the CSF proteome (A) Volcano plot comparing the CSF proteomes of LRRK2 G2019S versus WT carriers. The fold change in protein levels is depicted on the x axis and the –log10 t test p value on the y axis. Color scale is based on ANCOVA q values of the proteins differentially present in the CSF of LRRK2 G2019S carriers compared with the WT controls in the LCC cohort. Proteins with ANCOVA q values <5% are labeled. (B–D) HLA-DRA (B), PLD4 (C), and CTSS (D) protein intensity (log2) distributions in LRRK2 G2019S and WT carriers of the LCC cohort; p values of an unpaired t test are shown. Lines indicate mean and SD. (E) Annotation enrichment of GO terms using the LRRK2 G2019S versus WT fold changes (5% FDR). Terms with positive enrichment scores are enriched in the G2019S mutation over the WT and vice versa. (F) Heatmap of ANCOVA q values of all cathepsin proteins, which were quantified in both CSF and urine of LRRK2 G2019S carriers compared with the WT controls.

Given the substantial number of subjects carrying the *LRRK2* G2019S mutation in the LCC cohort, we explored whether the CSF proteome is altered by LRRK2 mutation status. We again applied an ANCOVA with sex, age at sample collection, study center, and PD status as confounding factors. At a FDR of 5%, the mutation significantly altered the abundance of the proteins HLA-DRA, HLA-DRB1, HLA-DPA1, CTSS, PLD4, TKT, ITGB2, PRDX3, ITIH5, CNDP1, and FAH (Figure 4A). On the basis of the peptides identified in the samples, we could distinguish two forms of the protein HLA-DRB1, which were both significantly enriched in LRRK2 G0219S carriers. Furthermore, Student’s t test with a FDR of 5% visually confirmed the upregulation of HLA-DRA, HLA-DRB1, and HLA-DPA1 in the mutation carriers (Figures 4A and 4B). These HLA proteins as well as PLD4 and CTSS were robustly quantified with at least four peptides in the LCC cohort, and their levels were significantly elevated in PD patients (>1.7-fold; Figures 4B–4D and Table S4). Using this approach globally, we observed that proteins elevated in the CSF proteomes of LRRK2 G2019S carriers compared with wild-type (WT) allele carriers were enriched for categories related to immune and inflammatory responses, further supporting the substantial evidence of a close association between enhanced inflammatory response and PD^43^^,^^44^ (Figure 4E).

Moreover, the analysis of non-manifesting LRRK2 carriers (NMCs) to healthy individuals also revealed several HLA molecules, CTSS as well as ADAM10 and FAM234A (ITFG3) to be upregulated in pathogenic LRRK2 carriers, with HLA-DRB1 being the only protein reaching the significance level at a FDR of 5% (Figure S2G). Pathogenic LRRK2 carriers have a higher likelihood of developing PD compared with healthy individuals; thus, proteins identified in this analysis may include prognostic factors.

Cathepsins are proteases mediating protein degradation and turnover in endolysosomal compartments, and several of them have been implicated in inflammatory diseases, lysosomal storage, and neurodegenerative disorders such as PD.^45^^,^^46^ We have recently shown that the levels of multiple members of the cathepsin family, including cathepsins A, B, C, D, H, L, O, S, and Z, significantly increased in the urine of LRRK2 G2019S carriers in two independent cohorts.^25^ We also detected these cathepsins in CSF but—in contrast to urine—only cathepsin S (CTSS) was significantly affected by the mutation status (Figure 4F).

### Integration of CSF and urinary proteome profiles

**Figure 5:**
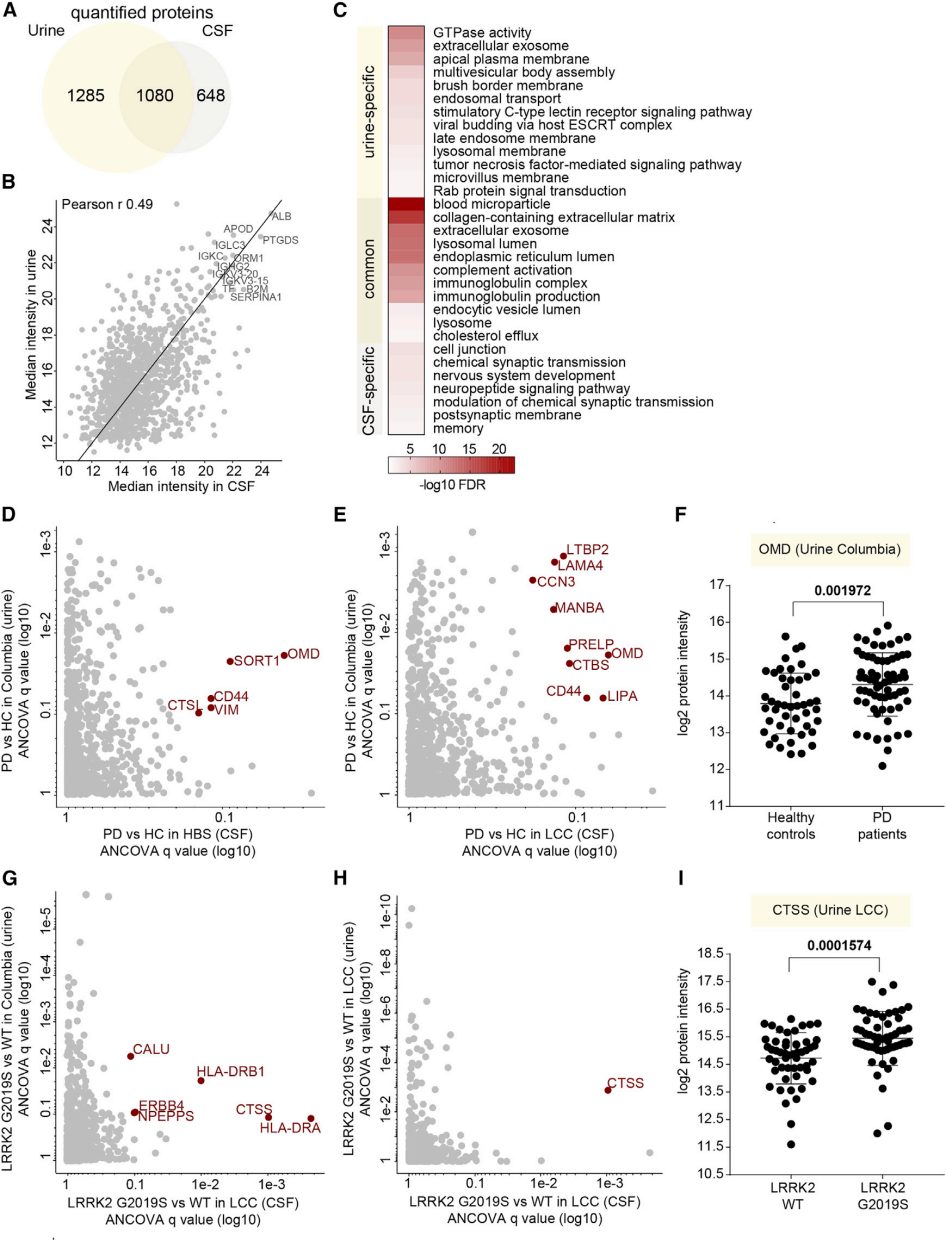
Integration of CSF and urine proteome profiles (A) Overlapping proteins between the CSF and urinary proteomes. (B) CSF-urine proteome abundance map based on median MS intensities of common proteins. Highly abundant proteins in both datasets are labeled as examples. (C) Fisher exact test to identify significantly enriched GO terms among the common and urine- and CSF-specific protein groups. Significant and non-redundant GO terms are displayed (FDR <5%). (D) Correlation of ANCOVA q values of the proteins differentially present in the CSF (HBS) and urine (Columbia) of PD patients compared with controls. (E) Correlation of ANCOVA q values of the proteins differentially present in the CSF (LCC) and urine (Columbia) of PD patients compared with controls. (F) OMD protein intensity distribution in the urine of controls and PD patients of the Columbia cohort. Results of unpaired t test are shown. The lines indicate mean and SD. (G) Correlation of ANCOVA q values of the proteins differentially present in the CSF (LCC) and urine (Columbia) of LRRK2 G2019S carriers compared with the LRRK2 WT controls. (H) Correlation of ANCOVA q values of the proteins differentially present in the CSF (LCC) and urine (LCC) of LRRK2 G2019S carriers compared with the LRRK2 WT controls. (I) CTSS protein intensity (log2) distribution in the urine of LRRK2 G2019S and WT carriers of the LCC cohort. Results of an unpaired t test are shown. The lines indicate mean and SD.

We previously analyzed 235 urine samples from two independent cross-sectional cohorts (Columbia and LCC), including two types of controls, healthy individuals, and LRRK2 G2019S carriers not manifesting the disease, and PD patients with and without the LRRK2 G2019S mutation, quantifying 2,365 urinary proteins in total.^25^ Encouraged by the great depth of the acquired urine and CSF proteomes in our previous and the present study, we decided to integrate both proteomes to determine co-regulated proteins. Although we analyzed both urine and CSF from subsets of the LCC cohort, there were no matching urine and CSF samples from the same individuals. More than 1,000 proteins overlapped, corresponding to 36% of all identified proteins in both biofluids (Figure 5A). Matching the common protein abundances revealed a clear correlation between the two biofluids (Pearson r = 0.49; Figure 5B). The most abundant proteins present in both included ALB, PTGDS, ORM1, SERPINA1, B2M, and several apolipoproteins and immunoglobulins (Figure 5B, labeled proteins at the top right). Moreover, Fischer’s exact test on the GO terms associated with the proteins specifically found in either urine or CSF or their common overlap revealed terms significantly enriched compared with all proteins identified in the two body fluids (Figure 5C). As expected, the terms related to nervous system including “postsynaptic membrane,” “memory,” “neuropeptide signaling pathway,” and “nervous system development” were enriched among proteins present exclusively in CSF. In contrast, terms related to the endosome-lysosome pathway as well as Rab protein signal transduction were enriched among urine-specific proteins, indicating that proteins of the Rab-LRRK2-pathway are highly abundant in urine, in line with our previous finding that the LRRK2 G2019S mutation strongly affects the urinary proteome.^25^

Correlating the ANCOVA q values of all common proteins quantified in CSF (HBS and LCC) and urine (Columbia cohort) of PD patients compared with the controls revealed several proteins regulated in both fluids (Figures 5D and 5E). Among those was osteomodulin (OMD), the level of which was also significantly elevated in the urine of PD patients, with a mean fold change of 1.47 (Figure 5F). Next, we integrated ANCOVA q values of LRRK2 G2019S versus LRRK2 WT of all common proteins quantified in the CSF samples of the LCC cohort and the urine samples of either the Columbia or the LCC cohorts (Figures 5G and 5H). This identified cathepsin S to be regulated in a LRRK2 status-dependent manner in both matrices (Figures 5G and 5H). The levels of cathepsin S were higher in the CSF of LRRK2 G2019S carriers compared with the LRRK2 WT carriers, with mean fold changes of 1.31 (p value of 0.0619) and 1.65 (p value of 0.0001574) in the Columbia and LCC cohorts, respectively (Figures 5I and S2H).

### Correlation of CSF proteome profiles with clinical scores indicating disease severity

**Figure 6:**
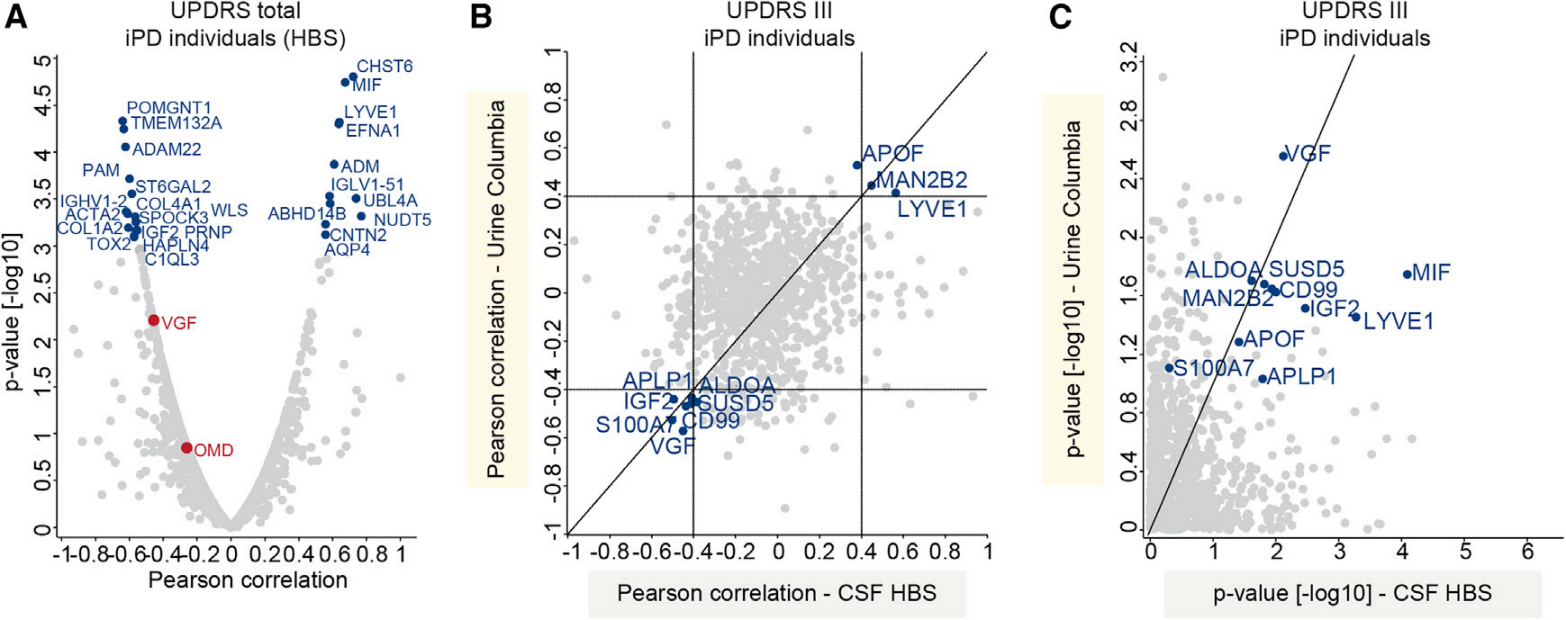
Correlation of CSF and urinary proteomes with UPDRS scores in idiopathic PD (iPD) patients (A) Correlation analysis of protein intensities in CSF with the UPDRS total scores in iPD patients. Pearson correlation coefficients and –log10 p values are displayed on the x and y axes, respectively. Proteins significantly correlating with UPDRS score (positively or negatively with a p value < 0.001) are labeled. (B) Correlation between Pearson correlation coefficients for correlation of urinary and CSF proteomes with UDPRS III scores. (C) Correlation between –log10 p values for correlation of urinary and CSF proteomes with UDPRS III scores. The proteins with Pearson correlation coefficients >0.4 and < −0.4 in both datasets are labeled in (B) and (C).

We next investigated whether any protein level changes in the HBS cohort correlated with the severity of PD pathology as assessed by the Unified Parkinson’s Disease Rating Scale (UPDRS) (Figures 6, S3, S4, and S5). The UPDRS scores and protein intensities are listed in Tables S1 and S2. We found 27 proteins to be significantly correlated with the UPDRS scores in idiopathic PD patients (p < 0.001; Figure 6A). Proteins showing the highest positive correlation in PD patients included CHST6 (p = 1.6E-5 and Pearson correlation r = 0.72), MIF (p = 1.8E-5 and r = 0.67), LYVE1 (p = 4.8E-5 and r = 0.64), EFNA1 (p = 5E-5 and r = 0.64), and ADM (p = 1.3E-4 and r = 0.61; Figure 6A). Proteins showing the highest negative correlation in PD patients included POMGNT1 (p = 4.6E-5 and r= −0.64), TMEM132A (p = 5.7E-5 and r = −0.63), ADAM22 (p = 8.8E-5 and r = −0.62), PAM (p = 1.9E-4 and r = −0.6), and ST6GAL2 (p = 2.7E-5 and r = −0.59; Figure 6A). Interestingly, none of these proteins was significantly altered in PD cases compared with controls. However, ADM was the strongest negatively correlated protein with the Mini-Mental State Examination (MMSE) scores (p = 9.3E-5 and r = −0.43; Figure S3D, a quantitative measure of cognitive impairment. Of note, no individual suffered from significant dementia or cognitive impairment at the time of sample collection (Figure S1D: average scores 28.3 in PD versus 28.4 in controls).

Furthermore, we compared the correlation scores of CSF proteins with those obtained in our previous study in urine. The comparison of Pearson coefficients for the UPDRS III scores (the only score available for urine) revealed 10 proteins (APOF, MAN2B2, LYVE1, APLP1, ALDOA, IGF2, SUSD5, CD99, S100A7, and VGF) to have coefficients either higher than 0.4 or lower than −0.4 in both matrices (Figure 6B). Among these, VGF exhibited a significantly negative correlation with the UPDRS III scores in both datasets (p = 2.8E-3 and 7.6E-3 in urine and CSF, respectively; Figure 6C). Overall, this analysis suggests that disease progression in PD patients, assessed by motor function, affects a similar set of proteins in CSF and urine.

Within the LCC cohort, Montreal Cognitive Assessment (MoCA) scores for all subjects were available (Figure S1I). Interestingly, we found CHIT1 to significantly negatively correlate with MoCA scores in all patients (p = 1.6E-5 and r = −0.48 in all subjects; Figure S5D), and this correlation was even stronger in PD patients (p = 1.9E-9 and r = −0.81 in PD patients; Figure S5E). Two proteins strongly positively correlated with MoCA scores, RELN and PENK, were negatively correlated with age in CSF (Figures S5D and S5E). Thus, multiple proteins in the CSF proteome correlate well with disease severity and different clinically important aspects of the disease.

## Discussion

Here, we applied a scalable and highly reproducible MS-based proteomics workflow to CSF samples from two independent PD cohorts. To the best of our knowledge, our approach resulted in the deepest single-run CSF proteome acquired by MS to date, with about 1,400 proteins quantified per sample. In addition, our workflow is sensitive, using only 40 μL of CSF for sample preparation. It does not require depletion of highly abundant proteins such as albumin or any biochemical enrichment, and the amount of purified peptides is sufficient for several MS runs, thus allowing the re-measurement of individual samples if required. Despite the unmatched depth of our dataset, we have not yet identified some of the known and well-studied PD-relevant proteins such as α-synuclein and neurofilament light chain, although both proteins were present in the LCC cohort library, and neurofilament light chain was also present in the HBS cohort library. The high dynamic range of protein abundances in CSF limits the sensitivity of MS-based proteomics compared with antibody-based assays that are frequently used to study these low abundant PD markers. However, the analytical variation of our assay, with a median CV of 19%, was much better than the biological variation, with a CV of median 46%. Although single-analyte antibody-based assays like ELISAs often have even lower technical CVs, we found our workflow well suited to study disease-related biological differences on a proteome-scale.

To reduce systemic biases in the analyzed samples and minimize the effect of pre-analytical variation, we performed a thorough quality assessment of every sample using our previously reported quality marker panels.^37^ This analysis flagged several samples of both cohorts as contaminated with erythrocyte-specific proteins, and these were excluded from further analysis. Samples with a high degree of contamination were restricted to two of the four study centers, and erythrocyte counts, which are often determined following CSF sample collection, did not correlate with the degree of proteome contamination by erythrocyte-specific proteins. This is presumably because intact erythrocytes, which are typically determined in clinical laboratories, are frequently removed by centrifugation before samples are stored. Contaminations in the proteome are, however, not caused by intact erythrocytes but by hemolysis. We also cannot exclude the possibility that different CSF aliquots were collected from each patient and that standard laboratory tests like erythrocyte counts were performed on only one of these aliquots. For the future, we recommend collecting CSF in bulk first and aliquoting only following thorough mixing. Our findings using untargeted proteomics clearly underline the importance of stringent quality control and study protocols to avoid systemic biases, which may result in seemingly significant regulations in some quality markers that would then be reported as potential biomarker candidates.

ANCOVA analysis considers confounding factors such as age, sex, or LRRK2 status and thus is well suited to stringently assess which protein changes are truly associated with PD status. This is important when analyzing cohorts with slightly imbalanced demographics between the compared groups like increased age of patients compared with controls, as frequently found in PD cohorts. When we employed ANCOVA to identify proteins that were disease-status-dependently regulated in both cohorts, OMD and CD44 stood out in both cohorts. OMD belongs to the small leucine-rich proteoglycans (SLRPs) and is involved in the organization and homeostasis of the extracellular matrix. CD44 is a cell surface glycoprotein involved in cell-adhesion and cell-cell interactions.^47^^,^^48^ Our results are in line with previous studies that identified OMD as a potential blood biomarker for PD^49^ and upregulated in a SNCA transgenic mouse model.^50^ A recent study has also shown the elevated expression of CD44 in the substantia nigra of human PD brains and CD44-mediated anti-inflammatory effects in primary mouse astrocytes.^51^ Induced expression of CD44 by α-synuclein in microglia, likely affecting PD pathogenesis by recruiting reactive microglia into the pathological region of the PD brain, was also reported.^52^ The low number of significantly regulated proteins in our study may be explained by the limited number of samples per disease group in each cohort combined with many potential confounders taken into account. Furthermore, patient heterogeneity due to inconsistent criteria for PD diagnosis and patient recruitment likely diminishes the overlap between independent cohorts. A recent meta-analysis also revealed that the overlap between independent studies is rather small and that future studies with larger-sample cohorts are required to identify protein changes with small effect sizes.^53^

A GO term enrichment analysis revealed that proteins upregulated in PD patients were associated with lysosome-related terms, which agrees with previous data that lysosomal dysregulation is evident in PD patients and involved in disease pathogenesis. In fact, lysosomal enzymes have long been investigated for their potential as biomarkers in the diagnosis of neurodegenerative disorders including PD.^14^^,^^54^, ^55^, ^56^, ^57^, ^58^ Changes in enzyme activities or abundances of lysosomal enzymes in CSF have been suggested to mirror the neuropathological changes linked to PD, although the basis of these alterations is not well understood. Levels of several lysosomal proteases including cathepsin D and cathepsin B are increased in CSF and *post mortem* brain tissue of Alzheimer’s disease patients.^59^^,^^60^ Furthermore, activities of the lysosomal β-galactosidase and β-hexosaminidase as well as cathepsin L in CSF or *post mortem* brain tissue of PD patients are elevated.^55^^,^^57^^,^^61^, ^62^, ^63^ Recently, we have shown that the pathogenic LRRK2-dependent changes of the urinary proteome included dozens of lysosomal proteins that could serve as biomarkers to stratify individuals with pathogenic LRRK2. In line with all these findings, we here identified two lysosomal proteins, cathepsin K and MAN2B1, among the most important features to classify an individual’s PD status based on their CSF proteome. CTSS, another cathepsin, was one of the proteins with the highest upregulation in CSF of LRRK2 G2019S carriers compared with the LRRK2 WT controls in the LCC cohort. While cathepsin K has the potential to ameliorate α-synuclein pathology by degrading α-synuclein amyloids,^64^ increased expression of the CTSS gene—together with other genes involved in the antigen processing and presentation pathway and related immune pathways such as HLA-DQA1, HLA-DRA, HLA-DPA1,and HLA-DMB—was reported in idiopathic PD patients in a study in which brain transcriptomic profiling was performed in idiopathic and LRRK2-associated PD.^65^ Moreover, cathepsin S has been shown to regulate the MHC class II antigen presentation process.^66^^,^^67^

Strikingly, several HLA proteins (HLA-DRA, HLA-DRB1, and HLA-DPA) were significantly increased in LRRK2 G2019S carriers compared with controls. The HLA locus is one of the key loci associated with susceptibility for PD.^68^ HLA-DR and HLA-DP are commonly expressed MHC-II molecules on antigen-presenting cells, including microglia in the CNS.^69^ Moreover, increased expression of HLA-DR is a hallmark of activated microglia, which are present in multiple neurodegenerative diseases including PD.^69^, ^70^, ^71^, ^72^ In addition, specific HLA-DRB1 variants can bind α-synuclein with high affinity, and genome-wide association studies identified HLA-DRB1 and HLA-DRA alleles to be associated with PD in different populations.^73^, ^74^, ^75^, ^76^, ^77^, ^78^ A recent study has revealed a positive correlation between LRRK2 and MHC-II levels in PD patients and a negative correlation in healthy controls.^79^ Our data support these findings and suggest a contribution of immunity and MHC-II molecules to the pathogenesis of familial PD.

Comparing the analyzed CSF proteomes with our urinary proteome profiles in PD patients revealed 1,080 common proteins. This large overlap and the clear correlation of the corresponding protein intensities (Pearson r 0.49) are remarkable and presumably due to the shared blood plasma origin of both CSF and urine. Interestingly, OMD was upregulated in PD patients in both CSF and urine, suggesting that the pathophysiology of PD affecting the OMD pathway is not restricted to the brain. Furthermore, CTSS was upregulated in LRRK2 G2019S carriers in both body fluids. LRRK2 is known to be ubiquitously expressed in many organs, and the observed dysregulation of lysosomal enzymes including CTSS may be due to the hyperactivity of the mutated kinase. Moreover, we found VGF to be important in classifying PD patients from healthy controls by ML. Strikingly, we also reported this in our previous urinary-proteome-profiling study where VGF was the most important feature for classifying LRRK2 PD patients from NMCs, and its levels were strongly decreased in PD patients.^25^ Consistently, VGF was also one of the handful of proteins found to be statistically significantly under-expressed in CSF of two independent PD cohorts.^28^ Its negative correlation with the UPDRS-III scores of PD patients in both CSF and urine could indicate a protective role of this growth factor for motor function. Despite these interesting observations, the number of samples analyzed in our cohorts is still low for ML approaches, and larger cohorts are needed to further improve the accuracy and generalizability of the extracted models. Nevertheless, our data show that CSF and urine have large overlaps in their proteomes and that similar PD- and LRRK2-associated proteome changes can be identified in both body fluids. Presumably, processes in both the CNS and distal organs contribute to the commonly observed regulations in the biofluid proteomes.

We also identified several proteins including CHI3L1, FCGR3A, NCR3LG1, and ZP2 (p <0.01 in all analyses and both cohorts) that correlate well with the subjects’ age at sample collection (Figures S3A–S3C and S5A-B). CHI3L1 is a secreted glycoprotein that serves as a migration factor for astrocytes and a marker of glial inflammation.^80^^,^^81^ Interestingly, CHI3L was reported to be upregulated in the CSF of Alzheimer’s disease patients and was therefore suggested as a biomarker for this disease. Its expression levels in various regions of the brain were shown to be correlated with age.^24^^,^^82^, ^83^, ^84^ Our data demonstrate that age correlates well with multiple proteins in the CSF and that it is thus important to consider these confounders in statistical analyses to avoid a biased interpretation.

In line with our finding of CHIT1 being negatively correlated with the MoCA scores, chitinases including CHIT1 have emerged as biomarkers in neurological disorders including amyotrophic lateral sclerosis (ALS), as their levels correlate with disease activity and progression, likely reflecting microglia/macrophage activation.^85^, ^86^, ^87^ In addition, levels of proteins such as CHST6 correlate well with disease progression, as measured by the UPDRS, especially UPDRS III (Figures 6A and S4). The sulfotransferase CHST6 plays an important role in keratan sulfonation, and mutations in the corresponding gene cause macular corneal dystrophy.^88^ Keratan sulfate, which is a type of sulfated glycosaminoglycan (GAG), is abundant in the brain, where it fulfills a multitude of functions.^89^ CHST6 expression is increased in the brains of AD patients, and its deficiency in mouse models mitigates AD pathology.^90^ Interestingly, the sulfation state of GAGs affects α-synuclein aggregation by regulating lysosomal degradation.^91^ Together, our data corroborate findings that sulfated GAGs like keratan sulfate can affect the pathophysiology of neurodegenerative diseases like PD. We also identified a putative correlation between UPDRS II scores and reduced LRPPRC expression, whose expression is also reduced in the blood of PD patients.^92^ Mutations in *LRPPRC* cause the early-onset progressive mitochondrial neurodegenerative disorder French-Canadian-type Leigh syndrome, characterized by defects in oxidative phosphorylation reminiscent to those found in prodromal PD.^93^

In conclusion, we have applied a highly reproducible and scalable MS-based proteomics workflow to perform proteome profiling of CSF in PD patients. We observed interesting proteome changes in PD patients and identified biomarker signatures that are specific to LRRK2 G2019S carriers. Further studies analyzing larger cohorts of patients will be required to confirm our findings and extend the panels of potential biomarkers. In a next step, clinical and targeted assays need to be developed to validate the biomarkers,^12^ followed by a test that can be used in clinical routine to enable early disease detection and patient stratification.

### Limitations of the study

The number of samples per disease group in each cohort in combination with patient heterogeneity and biological variation of the CSF proteome limits the power of the statistical analyses performed. Additional studies are needed to validate the identified biomarker candidates from this study using an independent cohort and potentially an orthogonal technology. Furthermore, clinical assays to measure these markers reliably in a routine fashion are still to be developed.

## STAR★Methods

### Key resources table

**Table:** 

REAGENT or RESOURCE	SOURCE	IDENTIFIER
**Biological samples**		

Human Cerebrospinal Fluid (CSF)	Harvard Biomarker Study	Healthy adult donors and Parkinson’s disease patients
Human Cerebrospinal Fluid (CSF)	Michael J. Fox Foundation for Parkinson’s Research (MJFF) funded LRRK2 Cohort Consortium (LCC)	Healthy adult donors and Parkinson’s disease patients, including LRRK2 G2019S carriers

**Chemicals, peptides, and recombinant proteins**		

Proteomics-grade modified trypsin	Sigma-Aldrich	Cat. no. T6567
Endopeptidase LysC	Wako Chemicals	Cat. no. 129-02541
Solid-phase extraction disks for SDB-RPS StageTips	Empore SDB-RPS	Cat. no.66886-U

**Critical commercial assays**		

PreOmics Lysis buffer	PreOmics GmbH	N/A

**Deposited data**		

Raw mass spectrometry data and Spectronaut output tables (identifier PXD02649)	This paper	https://www.ebi.ac.uk/pride/archive/

**Software and algorithms**		

Spectronaut (version 14.8.201029.47784)	Biognosys AG	https://biognosys.com/software/spectronaut/
Perseus (versions 1.6.0.7 and 1.6.1.3)	Tyanova et al., 2016 www.maxquant.org	https://maxquant.net/perseus/
GraphPad Prism (version 7.03)	N/A	https://www.graphpad.com/
MaxQuant Live software	Wichmann et al., 2019 www.maxquant.live	https://www.maxquant.org/mqlive/
OmicLearn (v1.0.0)	https://github.com/OmicEra/OmicLearn	https://share.streamlit.io/omicera/omiclearn/omic_learn.py
Python (version 3.7.6) using the pandas (version 1.0.1), numpy (version 1.18.1) and pingouin (version 0.3.4) packages	N/A	N/A

### Resource availability

#### Lead contact

Further information and request for resources and reagents should be directed to and will be fulfilled by the Lead Contact, Dr. Matthias Mann (mmann@biochem.mpg.de).

#### Materials availability

This study did not generate new unique reagents.

### Experimental model and subject details

In this study, CSF samples from two independent cross- sectional cohorts were analyzed. The first cohort consisted of 94 CSF samples from the Harvard Biomarkers Study (HBS) biobank and the second cohort was a subset of biobanked CSF samples from the Michael J. Fox Foundation for Parkinson’s Research (MJFF)-funded *LRRK2* Cohort Consortium (LCC). HBS and the proteomics analysis of HBS samples conducted in the current study were approved by the Institutional Review Board of Brigham and Women’s Hospital. The LCC study was established in 2009, when the MJFF LCC brought together investigators from North America, Europe, North Africa, and Asia to study individuals with mutations in the *LRRK2* gene. To be eligible to join the consortium, sites had to agree to share a core set of clinical data. Case report forms and standard operating procedures can be found at https://www.michaeljfox.org/news/lrrk2-cohort-consortium. Ethical review and approval was not required for the de-identified sample analysis in accordance with the local legislation and institutional requirements. The patients/participants provided their written informed consent to participate in this study. Disease severity was assessed by the Unified Parkinson’s Disease Rating Scale (UPDRS), which ranges from 0 for no impairment to a theoretical maximum of 199 for most severely affected individuals. UPDRS scores can be divided into four subscales for evaluating mentation, behavior, and mood (Part I, 0-16), activities of daily living (Part II, 0-52), motor examination (Part III, 0-108) and complications of therapy (Part IV, 0-23). Cognitive functioning was assessed using the Montreal Cognitive Assessment (MoCA), which ranges from 30 for no impairments to a theoretical minimum of 0 for most severely affected individuals. In addition, for the HBS cohort, Mini-Mental State Examination (MMSE) scores, which range from 30 to 0 (25-30 for normal cognition, 21-24 for mild dementia, 10-20 moderate dementia and 9 or lower for severe dementia) were also available.

### Method details

#### Sample preparation

40 μL of CSF samples were aliquoted in 96-well plates and processed with an automated set-up on an Agilent Bravo liquid handling platform.^16^^,^^24^ CSF samples were mixed with the equal amount of PreOmics lysis buffer (PreOmics GmbH) for reduction of disulfide bridges, cysteine alkylation, and protein denaturation at 95°C for 10 min. Upon 10 min cooling, 0.2 μg of each protease trypsin (Sigma-Aldrich) and LysC (Wako) was added to each sample (well) and digestion was performed at 37°C overnight. Peptides were then purified on two 14-gauge StageTip plugs packed with styrenedivinylbenzene- reverse phase sulfonate (SDB-RPS).^94^ Samples were first diluted with 1% trifluoroacetic acid (TFA) in isopropanol and loaded onto StageTips and subsequently washed with 200 μL of 1% TFA in isopropanol twice and 200 μL of 0.2% TFA/2% ACN (Acetonitrile). Peptides were eluted with 80 μL of 1.25% Ammonium hydroxide (NH4OH)/80% ACN, dried using a SpeedVac centrifuge at 45°C (Eppendorf, Concentrator plus), resuspended in 10 μL buffer A∗ (2% v/v ACN, 0.2% v/v tTFA, and stored at −20°C. Upon thawing before mass spectrometric analysis, samples were shaken for 5 min at 2,000 rpm (Thermomixer C, Eppendorf). Peptide concentrations were measured optically at 280nm (Nanodrop 2000, Thermo Scientific) and subsequently equalized using buffer A∗. 500 ng peptide was subjected to LC-MS/MS analysis.

Cohort-specific libraries were generated by pooling of 24 randomly selected samples of each cohort and separating the peptides of this pooled sample into 24 fractions each by high pH (pH 10) reversed-phase chromatography on the “spider fractionator”.^95^ Fractions were concatenated automatically by shifting the collection tube every 120 seconds. Upon collection, fractions were dried and resuspended in buffer A∗ for LC-MS/MS analysis. To increase the depth of each library, we isolated extracellular vesicles (EV) from pooled CSF samples of each cohort by ultra-centrifugation.^96^ Isolated EVs were resuspended in 100 μL of a sodium deoxycholate-based lysis buffer containing chloroacetamide (PreOmics GmbH), heated to 95°C for 10 min for reduction and alkylation and then digestion using trypsin and LysC enzymes at 37°C overnight. Peptides were desalted with SDB-RPS StageTips as described above. Peptides were eluted 80% ACN/5% NH4OH and the eluate was completely dried and resuspended in 0.1% formic acid (FA) for separation into eight fractions by high pH reversed-phase chromatography.^95^

To determine coefficients of variation, five aliquots of a pooled CSF sample on one plate were subjected to sample preparation (intra-plate) and three aliquots of the same pool were subjected to sample preparation on two different plates (inter-plate).

#### LC-MS/MS analysis

LC-MS/MS analysis was performed on a Q Exactive HF-X Orbitrap mass spectrometer with a nano-electrospray ion source coupled to an EASY-nLC 1200 HPLC (all Thermo Fisher Scientific). Peptides were separated at 60°C on 50 cm columns with an inner diameter of 75 μm packed in-house with ReproSil-Pur C18-AQ 1.9 μm resin (Dr.Maisch GmbH). Mobile phases A and B were 99.9/0.1% water/FA (v/v) and 80/20/0.1% ACN/water/FA (v/v/v). MS data for single-shot CSF samples were acquired using the MaxQuant Live software and a data-independent acquisition (DIA) mode with phase-constrained spectrum deconvolution.^97^^,^^98^ Full MS scans were acquired in the range of m/z 300–1,650 at a resolution of 60,000 at m/z 200 and the automatic gain control (AGC) set to 3e6, followed by two BoxCar scans with 12 isolation windows each and a resolution of 60,000 at m/z 200 were acquired.^99^ Full MS events were followed by 58 MS/MS windows per cycle in the range of m/z 300–1,650 at a resolution of 15,000 at m/z 200 and ions were accumulated to reach an AGC target value of 3e6 or for a maximum of 22 ms.

All fractionated samples including EV fractions were acquired using a top 12 data-dependent acquisition (DDA) mode. Full MS scans were acquired in the range of m/z 300–1,650 at a resolution of 60,000 at m/z 200. The automatic gain control (AGC) target was set to 3e6. MS/MS scans were acquired at a resolution of 15,000 at m/z 200.

#### Mass spectrometry data processing

The MS data of the fractionated pools and the single-shot CSF samples were combined into two cohort-specific hybrid libraries using Spectronaut version 14.8.201029.47784 (Biognosys AG). For all experiments except the machine learning with OmicLearn, the two cohorts were quantified separately. All searches were performed against the human SwissProt reference proteome of canonical and isoform sequences with 42,431 entries downloaded in July 2019. Searches used carbamidomethylation as fixed modification and acetylation of the protein N-terminus and oxidation of methionines as variable modifications. The Trypsin/P proteolytic cleavage rule was used, permitting a maximum of 2 missed cleavages and a minimum peptide length of 7 amino acids. The Q-value thresholds for library generation and DIA analyses were both set to 0.01. For individual protein correlations with clinical parameters and the machine learning, the Q-value data filtering setting in Spectronaut was set to “Qvalue”. For all other analyses, the setting was set to “Qvalue percentile” with a cutoff of 25%, to use only those peptides for the protein quantification that passed the Q-value threshold in at least 25% of all analyzed samples. The various runs were normalized to each other by using median intensities of common peptides.

### Quantification and statistical analysis

The Perseus software package versions 1.6.0.7 and 1.6.1.3^100^ and GraphPad Prism version 7.03 were used for the data analysis. Protein intensities were log2-transformed for further analysis apart from correlation and coefficient of variation analysis. Coefficients of variation (CVs) were calculated in Perseus for all inter-plate and intra-plate pairwise combinations of samples, the median values were reported as overall coefficient of variation. The protein CVs of the main study were calculated likewise within cohorts individually. For generation of the abundance curves, median protein abundances across all samples within a proteome were used. ANCOVA analysis was performed in python (version 3.7.6) using the pandas (version 1.0.1), numpy (version 1.18.1) and pingouin (version 0.3.4) packages. For the ANCOVA analysis, age at sample collection, *LRRK2* status (only in PD+ vs. PD-), and PD status (only LRRK2+ vs. LRRK2-) were set as confounding factors. The FDR (q values) was calculated using Benjamini-Hochberg correction. GO annotations were matched to the proteome data based on Uniprot identifiers. Annotation term enrichment was performed with Fisher exact test in Perseus separately for each cohort. Annotation terms were filtered for terms with an FDR of 5% after Benjamini-Hochberg correction in each cohort. Calculation of Pearson correlation scores and associated p values of protein intensities to UPDRS scores and other clinical parameters was performed in Perseus.

For machine learning, OmicLearn (v1.0.0) was utilized for performing the data analysis, model execution, and generating the plots and charts.^41^ Spectronaut output tables from the quantification analysis of both cohorts were used as the input for OmicLearn. No additional normalization on the data was performed. To impute missing values, a Zero-imputation strategy was used. Features were selected using ExtraTrees (n_trees = 500) strategy with the maximum number of 35 features. Normalization and feature selection was individually performed using the training data of each split. For classification, we used XGBoost-Classifier (random_state = 23 learning_rate = 0.3 min_split_loss = 0 max_depth = 15 min_child_weight = 1). We used (RepeatedStratifiedKFold) a repeated (n_repeats = 10), stratified cross-validation (n_splits = 5) approach to classify PD vs. HC, resulting in total 50 iterations for training the model, each time with 35 features. The average feature importance scores assigned by the classifier for each of the top20 features are shown in Figure 3G.

## Data Availability

- The mass spectrometry proteomics data have been deposited to the ProteomeXchange Consortium via the PRIDE partner repository with the dataset identifier PXD026491. - This study did not generate custom computer code. - Any additional information required to reanalyze the data reported in this work paper is available from the lead contact upon request.

## Supplemental information

Document S1. Figures S1–S6

Table S1. Summary of demographics of both cohorts

Table S2. List of identified proteins

Table S3. Quality marker panels

Table S4. ANCOVA and t-test statistics

Document S2. Article plus supplemental information
