# Supplementary material for: Proteome profiling of cerebrospinal fluid reveals biomarker candidates for Parkinson’s disease
Source: Cell Rep Med. 2022 Jun 21;3(6):100661. doi: 10.1016/j.xcrm.2022.100661 (PMC9245058; doi:10.1016/j.xcrm.2022.100661)
Supplement: Document S1. Figures S1–S6 [file mmc1.pdf]

Cell Reports Medicine, Volume *3*

Supplemental information

Proteome profiling of cerebrospinal fluid reveals biomarker candidates for Parkinson's disease

Ozge Karayel, Sebastian Virreira Winter, Shalini Padmanabhan, Yuliya I. Kuras, Duc Tung Vu, Idil Tuncali, Kalpana Merchant, Anne-Marie Wills, Clemens R. Scherzer, and Matthias Mann

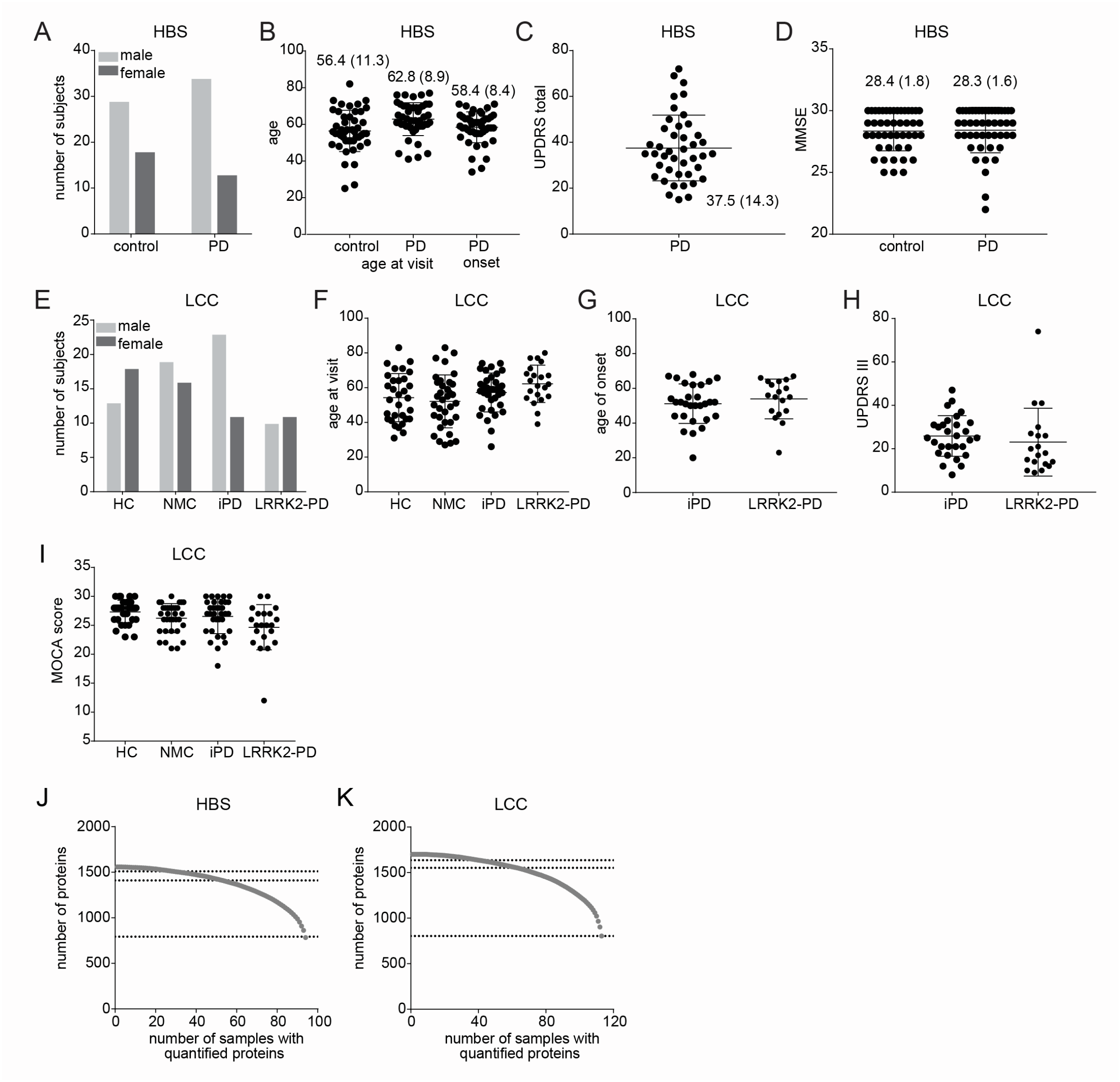

Figure S1. Clinical information and CSF proteome composition (Related to Table 1, Supplementary Table 1 and Figure 1)

A. Sex distribution among control subjects and PD patients in the HBS cohort. **B**. Age of subjects at the time of sample collection and disease onset for participants of the HBS cohort. **C.** UPDRS total scores for PD individuals of the HBS cohort. Mean and standard deviation are shown. **D.** MMSE scores for healthy and PD individuals of the HBS cohort. In B-D, means and standard deviations are given and also shown as lines. **E.** Distribution of subjects with different LRRK2 mutation status and manifestation of disease as well as sex in the LCC cohort. Number of subjects per group is shown. **F-G**. Age of subjects at the time of sample collection and disease onset for participants of the LCC cohort. **H.** UPDRS III scores for iPD and LRRK2 PD individuals of the LCC cohort. **I.** MoCA scores for all individuals of the LCC cohort. In F-I, lines indicate mean and standard deviation. **J-K.** Data completeness curves for each cohort. The number of proteins in the dataset (Y axis) depending on the minimum number of samples in which the proteins have each been quantified (X axis) is plotted. The dashed lines indicate 50%, 75%, and 100% data completeness.

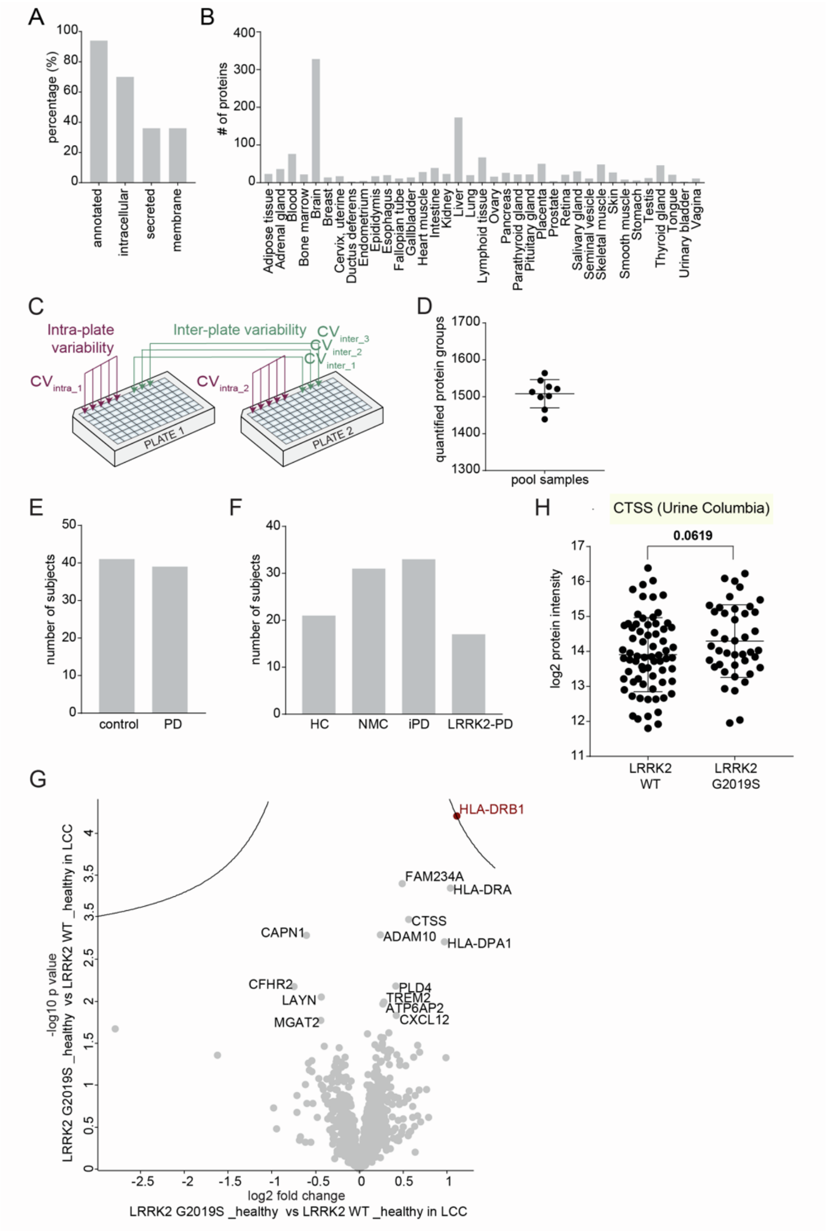

Figure S2. Assessment of the quantification precision and LRRK2-related alterations in CSF (Related Figure 1, 2 and 4)

A. Percentage of identified CSF proteins which are secreted, located inside of the cell or in the cellular membranes. Proteins are annotated based on the Human Protein Atlas database. **B.** Number of tissue-specific proteins identified in CSF. The information regarding tissue specificities is retrieved from the Human Protein Atlas database (the Tissue Atlas). **C.** Graphical overview of the experiment to determine coefficients of variation (CVs) for the quantification of CSF proteins. **D.** Number of proteins quantified in each sample for the CV determination experiment. The lines indicate mean and SD. **E-F.** Distribution of samples after filtering the poor-quality ones according to erythrocyte contamination in the HBS (C) and LCC (D) cohorts. **G.** Volcano plot comparing the CSF proteomes of healthy subjects carrying LRRK2 G2019S versus WT. The fold-change in protein levels is depicted on the x-axis and the -log10 t-test p-value on the y-axis. The line shows 5% FDR cut-off and a single significant protein is shown in red. **H.** CTSS protein intensity (log2) distribution in the urine of LRRK2 G2019S and WT carriers of the Columbia cohort. Unpaired t test is applied and the resulting p values are shown. The lines indicate mean and SD.

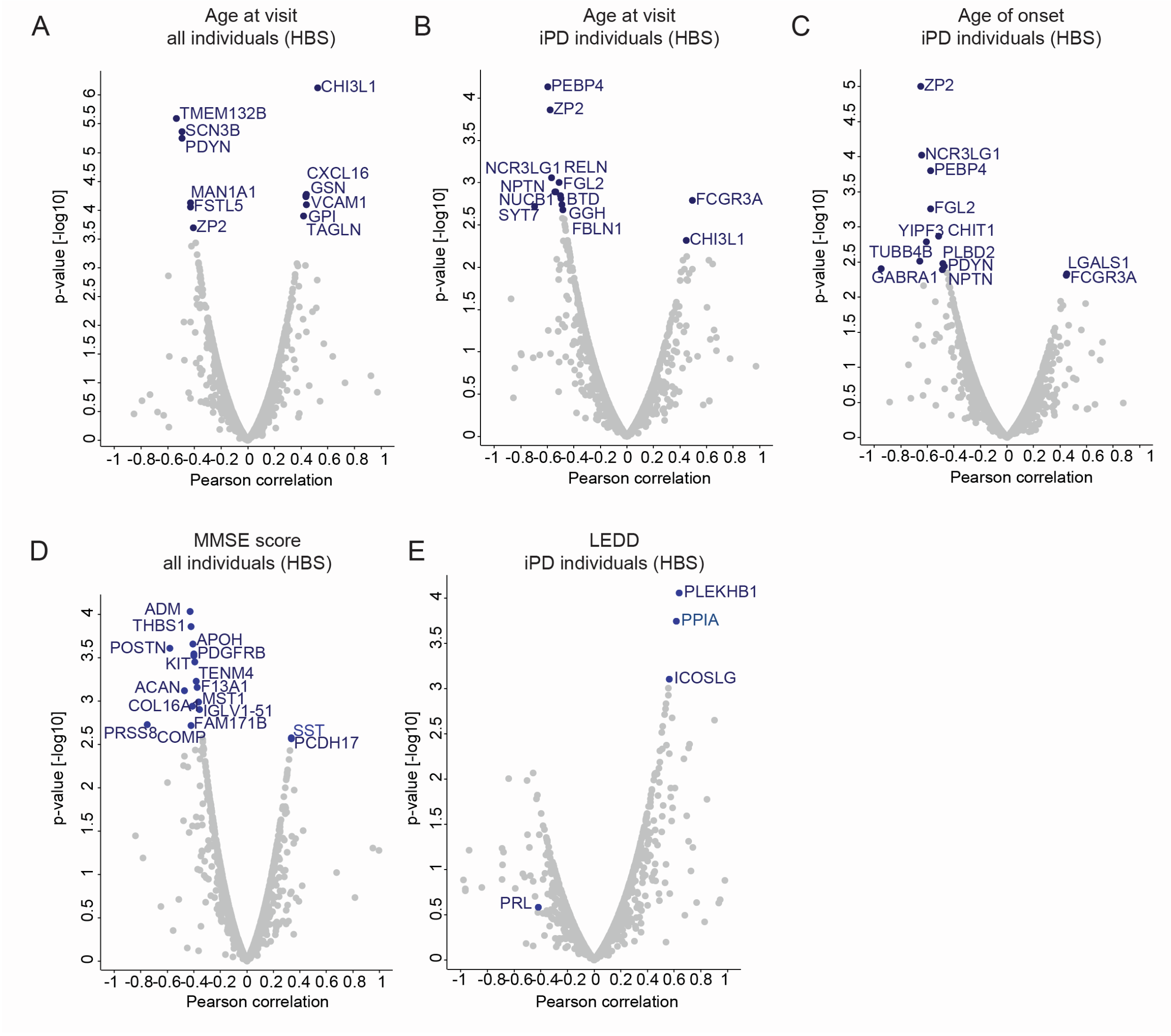

Figure S3. Correlation of protein abundances in the HBS with clinical parameters (Related to Table 1, Supplementary Table 1 and Figure 3 and 6)

A-B. Pearson correlation scores and associated p-values [-log10] of all protein intensities with the age at visit. All (A) or only iPD individuals (B) in the HBS cohort are included in the analyses as shown on top. **C.** Pearson correlation scores and associated p-values [-log10] of all protein intensities with the age of onset. All iPD individuals in the HBS cohort are included in the analysis. **D.** Pearson correlation scores and associated p-values [-log10] of all protein intensities with the MMSE score (Figure S1D). All individuals in the HBS cohort are included in the analysis. **E.** Pearson correlation scores and associated p-values [-log10] of all protein intensities with the LEDD levels. Only iPD cases in the HBS cohort are included in the analysis.

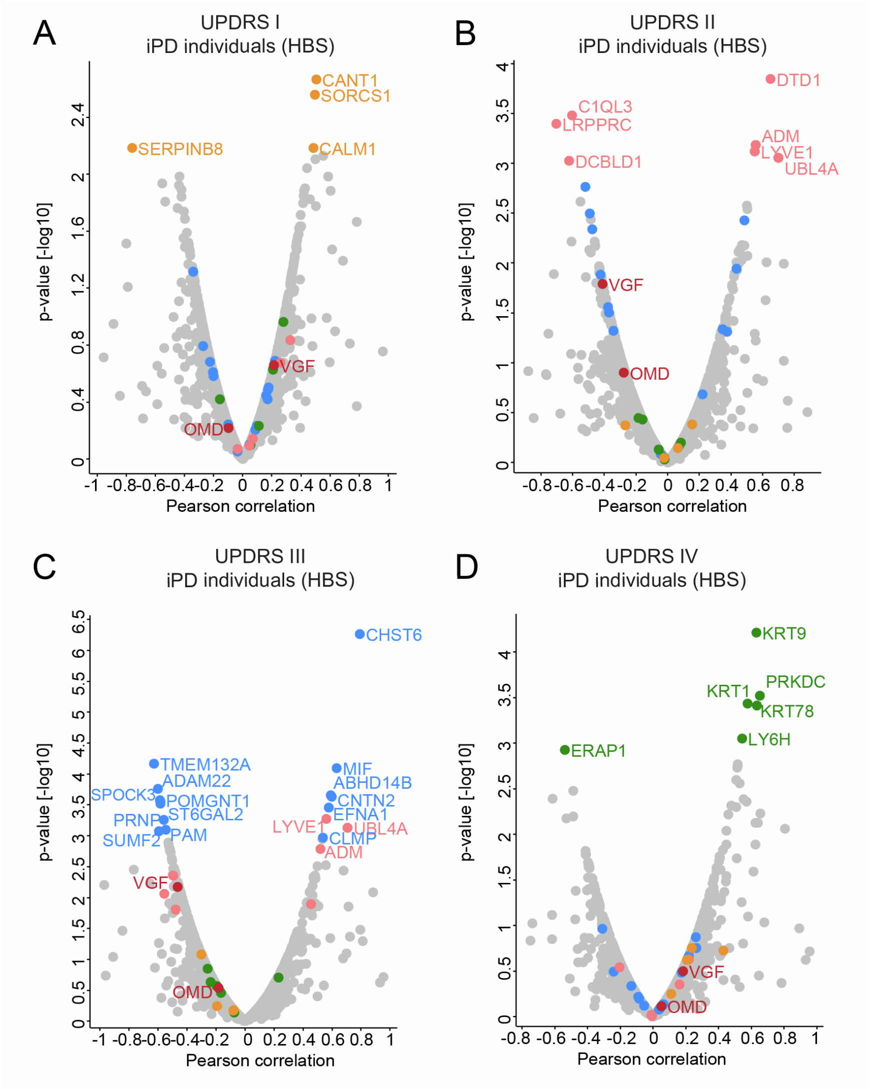

Figure S4. Correlation of protein abundances in the HBS with UPDRS scores (Table 1, Supplementary Table 1 and Figure 6)

A-D. Correlation analysis of protein intensities in CSF with the UPDRS Part I (A), Part II (B), Part III (C) and Part IV (D) scores in iPD patients separately identified a different set of significantly correlated proteins. Pearson correlation coefficients and -log10 p-values are displayed on the x- and y-axes, respectively. Proteins significantly correlating with UPDRS score (positively or negatively with a p-value < 0.001) are labeled.

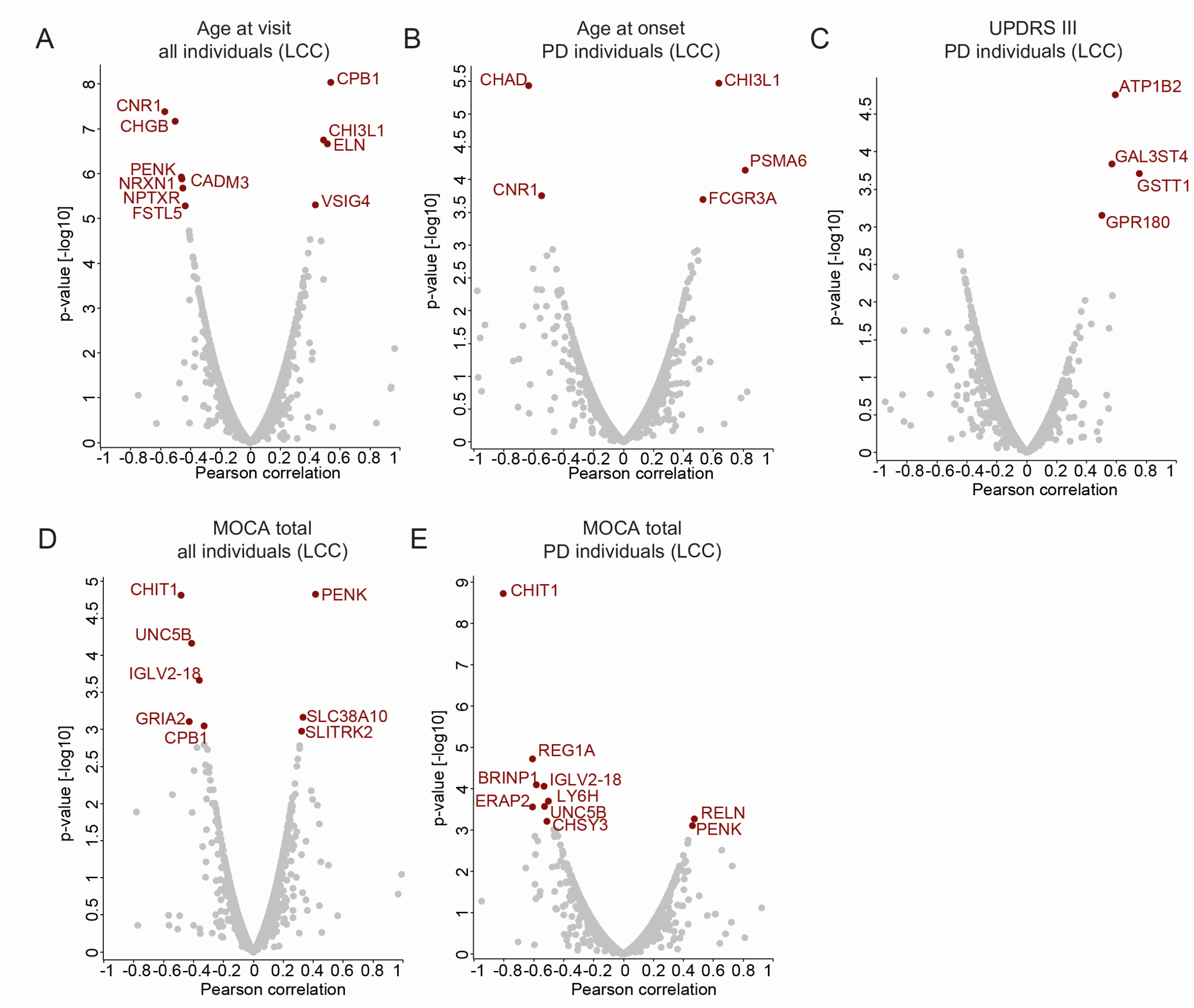

Figure S5. Correlation of protein abundances in the LCC with clinical parameters (Related to Table 1, Supplementary Table 1 and Figure 6)

A. Pearson correlation scores and associated p-values [-log10] of all protein intensities with the age at visit. All individuals in the LCC cohort are included in the analysis. **B**. Pearson correlation scores and associated p-values [-log10] of all protein intensities with the age of onset. PD patients in the LCC cohort are included in the analysis. **C.** Pearson correlation scores and associated p-values [-log10] of all protein intensities with the UPDRS III scores (ranging 8 to 74) for PD patients included in the analysis. **D-E.** Pearson correlation scores and associated p-values [-log10] of all protein intensities with the MoCA scores. All individuals (D) or only PD patients (E) in the LCC cohort are included in the analyses as indicated on top.

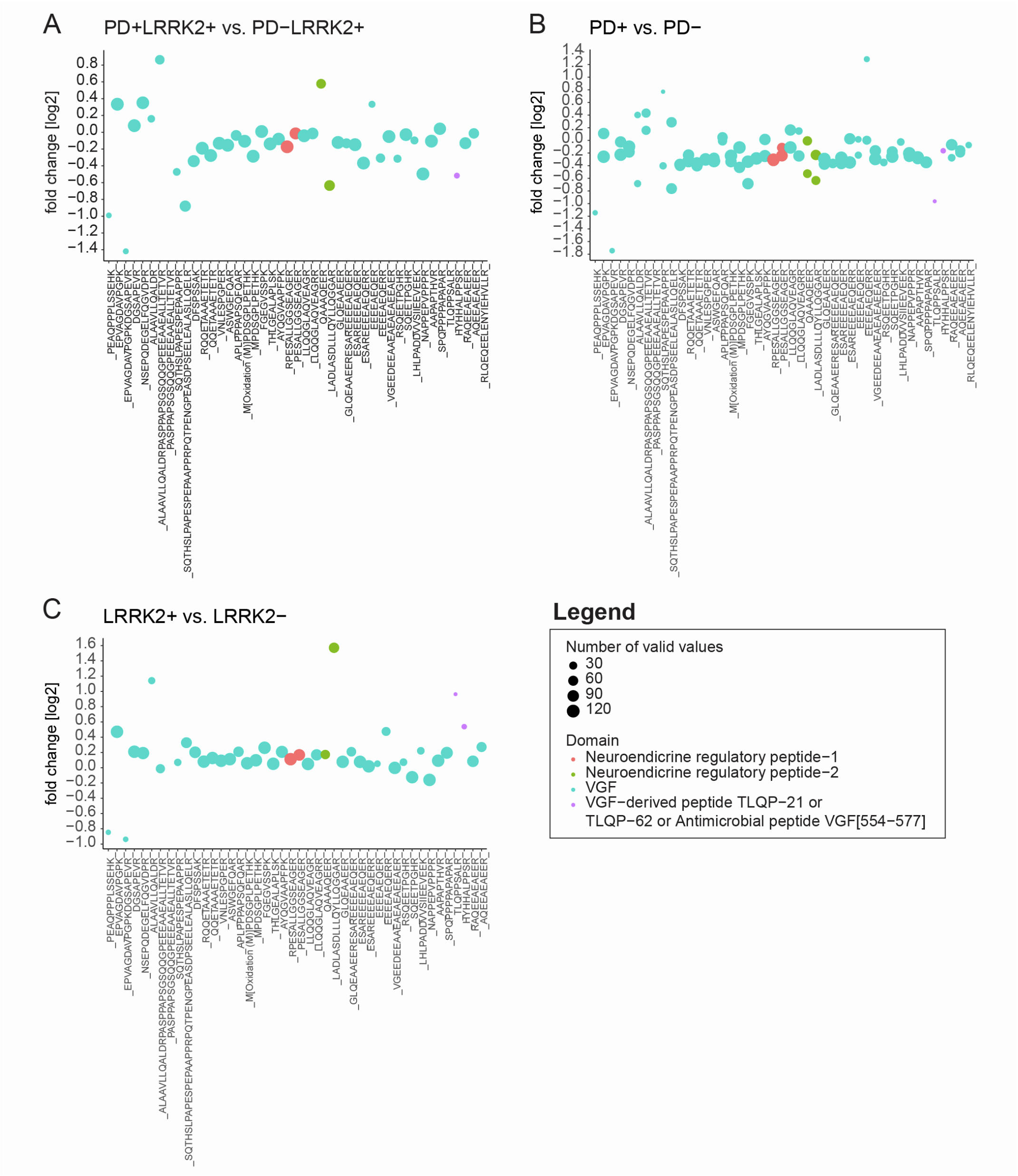

Figure S6. VGF peptides (Related to Figure 3 and 6)

A. Log2 fold-changes for each quantified VGF peptide comparing LRRK2+ PD patients with NMCs. The size of each dot represents the number of valid values (number of samples in which it was identified). The color indicates if the quantified peptide sequence is part of a known functional VGF peptide. **B**. Log2 fold-changes for each quantified VGF peptide comparing PD patients and controls. **C**. Log2 fold-changes for each quantified VGF peptide comparing LRRK2 G2019S carriers and WT allele carriers.
